# PIGMENTARY MOSAICISM: AN UPDATE

**DOI:** 10.4103/0019-5154.41658

**Published:** 2008

**Authors:** Rajoo Thapa

**Affiliations:** *From the Department of Pediatrics, The Institute of Child Health, Kolkatta, India*

Pigmentary mosiacism is characterized by cutaneous manifestations that are flat, hypo- or hyperpigmented lesions along the lines of Blaschko.^1^

Ever since Ito^2^ described the first case of hypomelanosis, which bears his name, it is understood that skin changes in hypomelanosis of Ito may be hypo- or hyperpigmentation. Flannery *et al.*^3^ hypothesized that the observed pigmentary abnormalities might be due to chromosomal mosaicism based on his observations of a girl with 45,X/46,Xr (Y)mosaicism in skin fibroblasts with pigmentary lesions.

Hypomelanosis of Ito and its varied clinical manifestations can be explained on the basis of chromosomal mosaicism. Mosaicism connotes that an individual is composed of two or more genetically different cell populations derived from a genetically homogeneous zygote, many of these representing postzygotic mutations. Chromosomal mosaicism is almost always demonstrated in cultured skin fibroblasts; blood lymphocytes are rarely affected. Aneuploidy including triploidy, trisomies, monosomies and structural abnormalities of autosomes such as rings, inversions and deletions, monosomies and structural abnormalities of sex chromosomes and X chromosomal translocations have been demonstrated. While there are reports of females having pigmentary mosaicism in the literature, only one report exists of a cell line with a structural abnormality involving an X chromosome. According to most workers, about one third of clinically diagnosed cases of pigmentary mosaicism demonstrate chromosomal mosaicism. This may be explained by the fact that the aneuploidy cell line may not be present in the tissues examined - a microdeletion may be present or a point mutation of a single gene present in the mosaic form. Moss *et al.*^4^ opined that the pigmentary pattern in the skin might reflect epidermal rather than dermal mosaicism. They cultured keratinocytes from four patients with pigmentary mosaicism: these patients had previously yielded normal chromosomal results in blood and fibroblast cultures, while in one patient, one out of 70 fibroblasts analyzed showed an abnormal chromosomal pattern. Keratinocyte culture confirmed this abnormal finding in this patient. Trisomy 7 was demonstrated in the keratinocytes of another patient whose fibroblast culture was negative.

Familial occurrence of pigmentary mosaicism has been found to occur at times. Jelinek *et al.*^5^ reported the case of a four year-old girl whose deceased paternal great-aunt was said to have had similar skin manifestations. Rubin^6^ described a female with hypomelanosis of Ito; her father and older brothers had depigmented areas in the skin. Groshans *et al.*^7^ reported a mother and her three daughters who were severely retarded with depigmented lesions on the trunk. The family history was consistent with an X-linked disorder and the histology reported was suggestive of X-linked dominant chondrodysplasia punctata rather than pigmentary mosaicism. Histopathological analysis of the hypopigmented areas on the skin demonstrated increased numbers of melanocytes and melanososmes in the basal layer of the epidermis.

Pigmentary mosaic disorders encompass multiple systems with phenotypic expression present mostly in the dermal and musculo-skeletal systems. Pigmentary abnormalities may take the form of either hypo- or hyperpigmentation and may be present at birth or may appear soon thereafter.^8^ The development of pigmentary lesions has been reported as late as two years after birth. Ultraviolet light may help detection in difficult cases. The edges of the hypo- or hyperpigmented areas are indistinct and close inspection shows variation in pigment within the broader, generally hyper- or hypopigmented lines.^1^

Hair abnormalities may consist of variations in color and texture (pepper-and-salt coloring) and alopecia. Nail changes reported are ridging and occasional hypoplasia/aplasia. Hypodontia, adontia, spacing irregularities and unusual dental cusps constitute the teeth abnormalities observed.^9^

Eye abnormalities are frequently reported. These include microphthalmia, iris coloboma, heterochromia irides and pinpoint pupils.^10^ Retinal pigmentary abnormalities with optic atrophy have also been observed.

Joint contractures, especially talipes,^11^ camptodactyly of fingers, asymmetric limbs and body parts are common. Other skeletal abnormalities include kyphoscoliosis, polydactyly, ectrodactyly, syndactyly and triphalangeal thumbs.^10^

Mental retardation of varying severity is also common.^9^ Seizures in association with neuronal migration disorders like pachygyria and heterotopias are occasionally seen. Autism may be seen in up to 10% of the patients.

Cardiac defects include ventricular septal defects;^8^ renal abnormalities comprise of unilateral renal agenesis and horseshoe kidneys.^11^ Genital defects consist of hypospadias and vaginal skin tags.

The author recently cared for a two year-old male child, born to nonconsanguineous parents who presented with an extremely unusual combination of hyperpigmented and hypopigmented skin lesions along the lines of Blaschko, sharply demarcated at the midline both in the anterior and posterior trunk regions. The child was diagnosed to have a combination of skin lesions comprising of hypomelanosis of Ito and whorled hypermelanosis.^12^

Any condition with skin manifestations along Blaschko's lines may be confused with hypomelanosis of Ito. Incontinentia pigmenti is marked by preceding vesicular or verrucous phases. Goltz focal dermal hypoplasia along Blaschko's lines may cause confusion but focal absence of dermis, multiple papillomas of mucous membranes and linear hypo- and hyperpigmentation suggest the existence of pigmentary mosaicism.^1^

No definite treatment for pigmentary disorders exists. Meticulous systemic examination should be carried out to detect additional abnormalities. Seizures should be managed with proper antiepileptics. Orthopedic and physical therapy should be instituted as and when required.

## References

[1] Donnai D, Harper J, Oranje A, Prose N (2006). Pigmentary mosaicism. Textbook of Pediatric Dermatology.

[2] Ito M (1952). Studies on melanin IX, Incontinentia Pigmenti achromians: A singular case of nevus depigmentosus systematicus bilateralis. Tohoku J Exp Med.

[3] Flannery DB, Byrd JR, Freeman WE, Perlman SA (1985). Hypomelanosis of Ito: A cutaneous marker of chromosomal mosaicism. Am J Hum Genet.

[4] Moss C, Larkins S, Stacey M, Blight A, Farndon PA, Davison EV (1993). Epidermal mosaicism and Blaschko's lines. J Med Genet.

[5] Jelinek JE, Bart RS, Schiff GM (1973). Hypomelanosis of Ito (‘incontinentia pigmenti achromians’): Report of three cases and review of the literature. Arch Dermatol.

[6] Rubin MB (1972). Incontinentia pigmenti achromians. Arch Dermatol.

[7] Grosshans EM, Stoebner P, Bergoend H, Stoll C (1971). Incontinentia pigmenti achromians (Ito). Dermatologica.

[8] Sybert VP, Pagon RA, Donlan M, Bradley CM (1990). Pigmentary abnormalities and mosaicism for chromosomal aberration: Association with clinical features similar to hypomelanosis of Ito. J Pediatr.

[9] Happle R, Vakilzadeh F (1982). Hamartomatous dental cusps in hypomelanosis of Ito. Clin Genet.

[10] Donnai D, Read AP, McKeown C, Andrews T (1988). Hypomelanosis of Ito: A manifestation of mosaicism or chimerism. J Med Genet.

[11] Thomas IT, Frias JL, Cantu ES, Lafer CZ, Flannery DB, Graham JG (1989). Association of pigmentary anomalies with chromosomal and genetic mosaicism and chimerism. Am J Hum Genet.

[12] Thapa R, Dhar S, Malakar R, Chakrabartty S (2007). Hypomelanosis of Ito-whorled hyperpigmentation combination: A mirror image presentation. Pediatr Dermatol.

